# A Motto for the New Year

**Published:** 1900-12-29

**Authors:** 


### A Motto for the New Year.

On the eve of a new year it is a wholesome exercise to make good resolutions. That such resolutions are often broken goes without saying, and that their crumbling remains pave the way to perdition is proverbial. Yet none the less is it a good and wholesome thing that as each year goes out we should resolve to do better in the next. All this, however, is commonplace, and has just been preached from a hundred pulpits. But if we turn to the practical side of our ordinary work, and if we consider for a moment how, under the pressure and strain involved in earning our daily bread, real scientific thoroughness in all we do is apt to be pushed aside, we cannot but admit that what is being hammered into us by the preachers in regard to "higher things" has a very serious bearing upon our professional avocation. It is not religion alone that is apt to be crushed down by worldly cares; science is in the same boat. The demon of inattention and inexactness is abroad, and we cannot suggest to our professional brethren a better motto for the new year than "Thoroughness in all things," and especially in observation and in diagnosis. Laxity in observation, and the omission of the daily duty of taking notes, are the two deadly sins, indulgence in which leads men down hill in the practice of their profession, leads to rule of thumb treatment, to pill and bottle practice, and generally to that indefinable, perhaps, but obvious lack of crispness of thought and of diagnosis which so often tempts the medical student, in the plenitude of his new-found knowledge, to look down as from a lofty pedestal upon the general practitioner and all his ways. Of course the medical student is very wrong and very presumptuous in so doing, but there is some truth in the view he takes of the wide difference between life in the wards and life in general practice, especially in relation to thoroughness of work. In the wards almost every case is one of interest and importance, but however commonplace any case may appear it has to be fully gone into and recorded. Thus is thoroughness encouraged, and a habit of mind is built up which regards every case as one demanding full investigation. In every-day general practice, however, the surroundings are often very different, and everything conspires to make the cult of thoroughness difficult and sometimes almost hopeless. Instead of the presumption being that each case is a "good" one there is every probability, considering the matter numerically, that it is an affair of but little interest; instead of the patient being pleased and gratified at the careful examination to which one submits him, he thinks we are but "making a job;" and instead of appreciating the trouble taken, he says he is in a hurry and only wants a bottle of physic. It is a thankless and often an almost hopeless task, and one need not be surprised that so many men fall short of the high intentions with which they entered upon practice. All the more need, however, for perseverance, and all the more necessity to begin the new year with the firm resolve to put behind one the demon of carelessness and inexactness.

Dr. Mitchell Bruce, a little time ago, speaking to the North-West London Clinical Society, urged this point with great force, saying that from mere lack of taking the trouble to investigate thoroughly, many cases of familiar diseases of the first importance are habitually overlooked in practice. It is not a question of failure to diagnose obscure cases after a carefully conducted investigation and deliberate consideration of every fact, but of failure to diagnose, or rather of failure to observe, perfectly obvious, unquestionable, and indeed familiar phenomena, which are staring us in the face all the time. The difficulty arises from being content with imperfect observation, being satisfied with an incomplete diagnosis, trusting to time to clear things up, allowing oneself to be so crushed by the multitude of "uninteresting cases" that the very habit of throughness is lost. We are in continual danger of falling into easy, slipshod, unmethodical ways, of feeling sure without having proper grounds for certainty, of accepting the most obvious explanation, and espepecially of giving way to the insidious temptation of regarding "diagnosis" as merely a guide to "treatment"—worthless unless it leads to some "practical" result. What then is the cure for this all-pervading vice of imperfect observation? The answer is—the taking of records, not of necessity written ones, but at least the keeping of a graphic record of what we observe.

"In routine practice," says Dr. Mitchell Bruce, "the record of physical signs and of some, at least, of the symptoms should be graphic—that is by means of diagrams and charts." Among the other good things that the use of clinical diagrams does is that it compels exactness. "Few of us will record a sign which does not exist, or map out an area or locate a tumour on a diagram made to scale unless we have faithfully ascertained it." Thus we come back to our point of departure. Definiteness of record compels accuracy of observation, and if we do but observe with sufficient care our diagnosis will not often go wrong. We wish all our professional brethren, and our professional sisters, too, A Happy New Year; and we hope that before the end of it their "case books" will be full and that their "diagrams" will be a record to be proud of.

